# Perception of the professional self-image by nurses and midwives. Psychometric adaptation of the Belimage questionnaire

**DOI:** 10.1186/s12912-023-01564-7

**Published:** 2023-10-31

**Authors:** Sabina Katarzyna Stadnicka, Danuta Zarzycka

**Affiliations:** 1 Departament of Obstetrics and Ginecology Didactics, Faculty of Health Sciences, Medical University of Warsaw, 02-091 Warsaw, Poland; 2 Departament of Pediatric Nursing, Ass. Prof, Medical University of Lublin, 20-093 Lublin, Poland

**Keywords:** Nurse self-image, Midwife self-image, Public image of a nurse, Public image of a midwife, Belimage

## Abstract

**Background:**

The aim of this paper is to present the research results on the perception of the professional self-image by Polish nurses and midwives as well as the psychometric adaptation of the Belimage scale.

**Methods:**

A cross-sectional survey was conducted from January to November 2018. The study group consisted of 670 clinical practice nurses and midwives. A diagnostic survey method was applied using the Belimage questionnaire after it obtained acceptable psychometric properties through an adaptation procedure.

**Results:**

In the group of respondents, the professional image of nurses and midwives is dominated by instrumental skills, documentation and organization of care, and communication skills. There is a statistically significant difference in the image of these two professions in terms of the ability to think and act creatively and critically, as well as innovation and evidence-based practice. The respondents' opinion regarding the perception of their image by themselves and society is statistically significant in each of the analyzed areas. In the professional image created by the public, nurses and midwives recognize that being a nurse/midwife is a vocation—277 (41.3%). However, in terms of their self-image, most nurses and midwives consider their work to be hard—442 (66%) and poorly paid—445 (66.4%). In the psychometric validation process, the Belimage questionnaire retained the original item structure, and the reliability of the subscales assessed with the Cronbach's alpha coefficient ranged from 0.845 to 0.730.

**Conclusions:**

The professional image of nurses and midwives varies depending on the profession and the perspective of the assessment in relation to themselves and society. The study showed a particularly unfavorable social image of nurses and midwives, which significantly influences the outlook of nurses and midwives themselves on this issue.

## Introduction

In the professions of public trust, such as the profession of a nurse and midwife, it is important to systematically monitor the social (external) and vocational (internal) image of the profession, in order to watch over its quality. Public opinion is an extremely effective instrument in the shaping of social standards and values. Nurses and midwives are health care professionals with extensive knowledge. However, the public opinion does not always appreciate the skills and competencies of nurses acquired through education and innovation. Despite the significant progress that has already been made in the profession, nurses and midwives still face big challenges related to their image, which affect their self-assessment and public image [1].

By monitoring the professional image, its professional diversity, inconsistency and sensitivity to many factors is indicated [2]. For example, in a debate on the social image of a nurse that took place in the Netherlands, attention was paid to the perception of a nurse through the prism of historical stereotypes as a woman who sympathizes with the patient and, at the same time, a doctor's assistant [3]. The issue of social stereotypes that determine the perception of the nursing or midwifery professions was established in an integrative review by a team of authors from Spain, who identified two categories, i.e. gender stereotypes of professionals and stereotypes of the profession itself. The nursing profession is perceived as a woman's profession with low qualifications, low social status, salary, academic and entry requirements, and little autonomy [4]. In Saudi Arabia, the nursing profession is not seen as a respected profession, as it was found, for example, that 71.5% of respondents would be ashamed to have a nurse in the family [5]. The negative image of the nursing profession is also found in other studies. Midwifery students most often perceive their future profession moderately positively, but these views change during the course of their education [6]. However, research from Ghana indicates a very negative perception of the midwifery profession due to a lack of respect for patients and aggressive care provided by midwives [7]. Therefore, in some contexts, significant efforts are required to increase public awareness of the roles and responsibilities of nurses and midwives. The World Health Organization (WHO) has declared 2020 the International Year of the Nurse and Midwife to recognize their contribution to global health care and to highlight the potential of their capabilities [8]. In 2019, Poland celebrated a half-century of academic training for nurses, which prompted an analysis of changes in the image of nursing and nurses. The detailed analysis showed a gradual decrease in the importance of empathy and caring in the perception of the nursing profession in favor of manual dexterity, use of specialized procedures, use of complex apparatus and investment in postgraduate education [9].

## Background

### Image of nurses and midwives

The nursing profession has undergone tremendous change over the past half century, particularly in terms of professionalization. Historically, nurses and midwives have been perceived as displaying integrity and a sense of professionalism [10].

A nurse's image is the way that the profession appears to others or the impression that it makes on others [11]. According to Fletcher (2007), the way nurses perceive themselves is defined as self-image. General appearance and behaviours define the image of a nurse and significantly contribute to building the brand of the profession certainty [12, 13]. An exploratory qualitative study isolated the structure of the image of nurses and midwives, which took into account the physical appearance of staff, communication strategies and behaviors, approach to work and professional competence [14]. The public image seems to be closely associated with the nurse self-image. This creates limits which restrict and construct the image of nursing [12].

Nurses do not have a very positive professional self-image, or sense of self-importance. A stereotypical and disparaging image is attributed to nurses around the world [15]. A Swiss study has demonstrated that nurses have internalised social stereotypes, and they sometimes use them themselves to explain their profession to others [16]. Individually, each nurse has the power to shape the nursing image. A negative self-image of nurses and midwives facilitates and reinforces the shaping of their unfavourable image in society [15].

### Image of nurses and midwives vs. the media

The media shape the image of the world, which does not strictly depend on the assessed reality, but a reflection of it conditioned by the beliefs of society [17].

The image of a seductive nurse, presented as a young, attractive, sensual and provocative woman, used only for her decorative appearance is often publically promoted [18]. An analysis of publications in the press, social media and videos identified four main perceptions of the profession: the image of nursing as a questionable profession; the entertainment value that demeans nurses; role inconsistency—nurses are trusted but not respected; and poor understanding of the role of a nurse [19]. As a consequence of the fact that nursing is dominated by women and the media industry is dominated by men, the process of changing the image of a nurse to adapt it to reality is a historically slow process [20]. Publications celebrating International Nurses Day were mainly positive and appeared in weekly community publications in May and June, rather than in national and regional daily newspapers. When the commemorative publications were excluded from the analysis, most of the articles unfortunately portrayed nursing in a negative way [21]. Although many efforts have been made over the years to rectify this situation, and the profession has undergone a significant and continuous evolution in the public consciousness, these stereotypes are still perpetuated by the media today [22].

The media should portray nursing in a positive light to create a positive image of the profession, reduce nurse shortages and improve the quality of health care [23, 24]. Whether young people choose to pursue nursing as a career or not depends on the image of the profession in the media and in the public eye [25]. A media representation policy is an important tool that influences the efforts of nurses. Nursing should be portrayed accurately and positively in the media, as images of nursing in the media are rooted in society [20]. Social media can be used to combat stereotypes in order to help the public better understand what nurses actually do [26]. Examples of best practices in changing the image of nurses and midwives include the *Nursing Now* and *Nursing Now Challenge* campaigns, which were successful in generating the first global social movement in nursing that led to the construction of a more effective narrative that is disseminated in the media as well as heard and appreciated in political decision-making processes [27].

The image of nursing should be updated to emphasize nurses' knowledge, skills, and expertise, and to recognize their multiple roles and tasks as well as the independent nature of the profession to both attract new employees and prepare them for the growing complexity of healthcare [25]. The future image must affirm that nursing is a profession "based on science, technology, and knowledge" [28].

### The image of nurses and midwives and health crises

It is worth noting that the image of nurses and midwives changes during global health crises, i.e. epidemics, pandemics, wars [28]. The current image was formed in the early days of the nursing profession around the service of nurses during conflicts and wars. Violent war metaphors often revolve around these images: fighting battles, fighting for patients, fighting diseases, working in the trenches, carrying out doctors' orders. These metaphors have created an image of nursing in which the nurse is seen as loyal, obedient, caring, servile and following orders. In addition, the nurse is also seen as compassionate, caring, nurturing, protective and maternal [29].

During the 1918 flu epidemic in Spain, articles appeared in the press that addressed aspects of the recognition of nurses' qualifications, the creation of nurses' professional profiles and early academic courses in nursing, as well as nurses' salary demands, strikes and the care offered by them [30]. An examination of the media's portrayal of nurses and their role during the 2014–2015 Ebola virus outbreak in West Africa revealed a lack of public awareness of nurses' crucial role in health care. Little attention was paid to nurses, their knowledge and experience. During the epidemic, the perception of nurses evolved from positive to negative [31]. These images still remain as the dominant images that the public thinks of when they think of nursing: people characterized by integrity, warmth and humanity [29]. In recent years, the media has portrayed nurses, midwives and other health care workers as heroes and told stories of their sacrifices, as the Covid-19 pandemic brought special attention to the public perception of health care [3, 32]. The majority of respondents believe that the pandemic has had a positive impact on the image of nursing [33]. Public opinion on social media seems to portray an image of nurses that reflects the professionalism and values of the profession [34]. The public and media profile of nursing has never been so high [35]. Therefore, we see the pandemic as an opportunity to sustain the growing sense of self-worth of nursing and strengthen confidence in a nurse's professional identity [36]. However, there is also still a "care gap" between nurses, midwives and other health care professionals regarding the role of a nurse, which can also relate to the image of the nursing profession. This gap concerns, for example, the expansion of a nurse’s ability to prescribe and administer medications, which is not accepted, especially by doctors. Therefore, the profession of nurse and midwife is often perceived as a subordinate profession that depends on doctors without autonomy in decision making [37, 38].

The negative image of a nurse can lower the motivation and self-confidence of those in the profession. In addition to this, it can negatively affect all plans and behaviors of members of the profession [39]. How nurses perceive their professional image affects their sense of professional self-esteem and motivates-positive or demotivates-negative for action. It is important to continue to study which factors most influenced and changed the image of nursing during the pandemic, and to develop strategies that would perpetuate a better image of nursing in society on an ongoing basis [29, 33]. The key to changing the image is to first envision the future state. Together, national nursing and midwifery organizations must reach a consensus on this new image in order to effectively implement their own specific statutes and influence the culture of nursing. The future image standard should include nurses/midwives as scientists, economists, entrepreneurs, epidemiologists and anthropologists.

We need to move from philosophizing about culture change to activism, because our success is based on how quickly we can adapt to a rapidly changing environment if we are to take advantage of the opportunities the pandemic has provided for our profession [28]. As stated in the literature, *“we cannot expect outsiders to be the guardians of our visibility and access to public media and health policy arenas, but we must develop the skills of presenting ourselves in and to the media” *[11].

### The status of nurses and midwives

In 2018, the number of certified nurses worldwide is just under 28 million, and the gap based on need is 5.9 million [5, 40, 41]. In order to address the shortage of nurses, which according to the World Health Organization will reach 9 million worldwide by 2030, with a deficit of 590,000 nurses in Europe alone, the number of nursing and midwifery graduates must be increased and they must be encouraged to stay in the profession (2021 State of the World's Nursing 2020). An analysis of The State of the World's Midwifery 2021 [SoWMy 2021] indicates the current global situation of health care workforce shortages. The shortage of all types of workers based on need is 1.1 million. But the largest shortage (900,000) is for nurses and midwives. It is projected that 82% of the population's needs can be met in 2030 (The State of the World's Midwifery 2021). In Poland, a report by the Head Chamber of Nurses and Midwives [NRPiP, 2021] identified numerous staffing shortages. The number of registered nurses is 232,387 and midwives 28,444, with more than half of them over the age of 50. Estimates indicate that by 2030, the number of employed nurses and midwives will decrease by 36,293.

Numerous reforms in the education system and changes in the organization of health care create new opportunities for nurses and midwives to develop their professional practice, become independent and raise their professional prestige by breaking the past historical images of these professions. The modern nurse and midwife should respond to the dynamics of changing expectations and needs of modern patients and society [42]. The profession of nurse and midwife in Poland have the status of an independent medical profession. The rules and conditions for practicing the profession of nurse and midwife are regulated in the basic legal act, the Act on the Profession of Nurse and Midwife. The Act defines the rules for: practicing the professions of a nurse and midwife separately; obtaining the right to practice as a nurse or midwife, professional training and postgraduate education. [Act of July 15, 2011 on the professions of nurse and midwife (Journal of Laws No. 174, item 1039)]. In addition, the two professions have the Act on the Self-Government of Nurses and Midwives of July 1, 2011, and the Code of Professional Ethics for Nurses and Midwives of the Republic of Poland in common. [Appendix to Resolution No. 18 of the National Congress of Nurses and Midwives of May 17, 2023.]

The level of qualification of nurses and midwives is the result of two subsystems of education. Between 1990 and 2005, the right to practice as a nurse or midwife could be obtained after completing a medical high school (five years), a medical post-secondary school (two years), a medical vocational college (two-and-a-half years), a medical vocational college (three years), or undergraduate study (three years). Since 2007, in accordance with the educational systems in place in countries that are members of the European Union, the study of nursing and midwifery is conducted in the form of higher education: bachelor's and master's degrees. [Strategy for the development of nursing and midwifery in Poland. Warsaw, December 2017.]

### Professionalism of nurses and midwives

Professionalism includes a set of attitudes, skills and behaviours, attributes and values expected of those considered experts by society. The professionalism of a nurse and midwife is an inevitable, complex, diverse and dynamic process [43, 44]. Currently, discussions in nursing circles concern the foundations of professionalism, which until recently were based on vocation, and currently, above all, on education [45]. Basic professional values may help normalise the profession and show the value of nursing to society [42]. In accordance with the evolutionary concept analysis by Rodgers (2018), three equally important elements of nursing professionalism have been distinguished:

- cognitive – focusing on the constant study of professional behaviour and the use of this knowledge in the work environment;
- attitude – including attitudes and ideas guiding nurses during the performance of their professional tasks and career development;
- psychomotor – promoting building of practical skills and sharing experience [43].

Given that the meaning of professionalism varies depending on time, context and culture, it is difficult to define a universal professional image of a nurse or midwife. The study of one of the newer concepts of nursing professionalism indicates its three attributes: multifactorial (knowledge, values, behavior), dynamic (socialization process, interaction process), culture-oriented (including religion) [24].

A good/positive professional image of nursing/midwifery is essential to counteract the looming staffing shortage. A strong image of the brand of nursing can dispel outdated and misguided perceptions while communicating new visionary leadership that aligns with the priorities of the nursing and midwifery profession [45].

Quite often, as studies show, professionalism of Polish nurses and midwives focuses on the professional activity depending on the doctor's decisions, i.e. diagnostic and therapeutic decisions relying on manual skills and expected qualities, such as speed, efficiency and effectiveness in action.

In addition, professionalism of nurses means effective and therapeutic communication, and always finding a way to act as part of the nursing care, also under the pressure of time [46].

### Framework

The image of nursing influences nurse recruitment, retention, recognition, value, and ultimately the health and well-being of individuals, families, and communities [25]. The image of nursing is one of the factors that influence career choices [47].

The negative image of a nurse can lead to a number of undesirable consequences, such as workforce shortages, impediments to interdisciplinary relationships, violence, public trust, low salaries, and high workloads and burnout [48, 49]. Nursing students' perceived image of nursing directly affects enrollment rate, graduation rate and employment rate within 5 years [50, 51].

Research on the professional image of Polish nurses and midwives was based on elements consistent with the adopted theoretical assumptions of M. Rosenberg (2018) [52] that answered the following questions:

- What is the reliability and theoretical accuracy of the Polish version of the Belimage scale?
- What are the differences in how nurses and midwives perceive of the image of their profession?
- What are the differences in the perception of nurses/midwives' own image and the social image of nurses/midwives?
- What are the differences in the perception of the level of satisfaction with the image of nurses/midwives and satisfaction with the social perception of the image of nurses/midwives?

### Aim

The purpose of this study is to present the opinions of the surveyed nurses and midwives on the current image of their profession in terms of similarities and differences in the image of nurses and midwives, and to compare the public and their image of qualified nurses and midwives in Poland.

As a prerequisite, an adaptation of the Belgian Belimage questionnaire was made before proceeding with the study proper.

## Materials and methods

The sample size was calculated based on the number of nurses and midwives with a current license to practice. In 2018, according to the Central Register of Nurses and Midwives (CRPiP), the number of registered nurses and midwives was 333,796, which included 295,481 nurses and 38,315 midwives (Report of the Head Chamber of Nurses and Midwives 2023). A significance level (α) of 95% and an acceptable error rate (e) of 5% was assumed, so the required sample size for the population of nurses and midwives was 384 people.

The research was conducted from January to November 2018 among nurses and midwives of clinical practice in 3 specialized hospitals located in different parts of the Central European country.

A representative sample of study sites was obtained with the use of the following inclusion criteria: geographical location: one hospital each in the eastern, middle and western regions, type of hospital: university, number of beds: > 600, and management structure: public. The inclusion criteria also required that the respondents be registered nurses or midwives according to Polish law, have direct contact with patients most of their working time, or occupy positions of clinical nursing supervision. Nursing assistants and medical caregivers were excluded due to their different type of education (post-secondary school or 2 semesters of Qualified Vocational Course until 2021). The exclusion criterion also included different professional competencies than those of nurses and midwives, as well as a different scope of professional autonomy. 750 nurses and midwives of clinical practice participated in the study, with a return of 680 surveys. The survey return was 90.66%. The studies were conducted using the Pen-and-Paper Personal Interview (PAPI) method. After checking all of the questionnaires and rejection of incomplete ones, 670 questionnaires were subject to statistical analysis. The study participants were instructed to define their self-image and how the public opinion perceives nurses and midwives, and what a professional image of persons representing these two professions should be, by answering the questions of the Belimage questionnaire. The research instruments were delivered and personally collected by the respondent. The participation in the study was anonymous and voluntary. Filling of the questionnaire constituted consent to the study participation and took place outside the place of work of the respondents.

### Measures

Belimage is a Belgian Professional Self-Image Instrument for Hospital Nurses and Midwives. The decision to choose this research tool was based on its European origin, because cross-border studies have shown significant differences in the image of nurses from different cultures, and research methods most often do not take into account cultural differences [53]. This instrument is intended for the self-assessment of the professional image of nurses and midwives of clinical practice. It consists of 40 questions covering 5 areas: personal information, education and professional competencies, nursing care, team care, and the context of nursing care (work environment). The questions are directed to nurses and midwives with various seniority and on various positions, starting from unit nurses/midwives up to head nurses/nursing directors [54, 55].

A consent to non-commercial use of the instrument was obtained from its co-author representing the BELIMAGE Group, Kristel De Vliegher, who has expressed written approval in electronic form regarding the use of the tool, its translation and adaptation to Polish conditions.

### Cross-cultural adaptation process

Cross-cultural adaptation and psychometric validation of Belimage was performed according to the World Health Organization (WHO 2016) schedule, which was implemented in the following stages:

- I: obtaining the authors’ consent to the cross-cultural adaptation and psychometric validation of the Belimage questionnaire
- II: translation of the scale from English to Polish by two independent translators (forward translation) – both translators graduated from English Philology and worked at higher education institutions, translating and teaching English;
- III: development of the Polish version of the Belimage questionnaire by a panel of experts. The expert panel consisted of 7 people: 5 nurses and 2 midwives. 3 people had primary education, while 4 people had higher education, one of them had a doctoral degree. The criteria for inclusion in the panel were a minimum of 5 years of work experience in the profession, possession of a license to practice as a nurse or midwife, Polish citizenship, current practice of the profession in direct care. As a result of the analyses, the nursing and midwifery specializations differing in the two countries, i.e. Belgium and Poland, were verified, the graphical compatibility of the tool, the number and formulation of questions and the forms of answers to the questions asked were maintained. In the above issues, the concordance of expert opinion ranged from 82 to 100%;
- IV: so called “back translation” from Polish to English was performed, and the two versions of the scale were compared; the retranslation of the scale back to English was performed by a translator whose native language is English but who is also fluent in Polish. Both versions of the translation revealed no significant differences.
- V: determining the final version of the Belimage questionnaire, the communicability of the questionnaire items was assessed by 5 experts who were a nurse and midwife with a master's degree, a senior nurse/senior midwife and a nurse with a doctoral degree in health sciences.
- VI: conducting an evaluation of the psychometric properties and structural accuracy of the final version of the Belimage questionnaire.

### Ethical consideration

The study design was approved by the Bioethics Committee, which was contained in the opinion with ref. no. KE-0254/82/2018. The participation in the research was voluntary and anonymous. The authors declare that the study was conducted in compliance with the ethical principles included in the declaration of Helsinki.

### Data analysis

Statistical analyses were performed with the use of the Statistical Package for Social Sciences (SPSS, IBM, Stanford, CA, USA).

Basic descriptive statistics were determined for all measurable (quantitative) parameters: mean, median, standard deviation, and for qualitative parameters: frequency and percentage. The analysis of the normality of variable distributions was performed using Kolmogorov-Smirnov tests. The analysis of the equality of groups was performed using the Pearson Chi-square test. In order to isolate the internal structure of the scales, an exploratory factor analysis with Varimax rotation was performed, and if the internal structure of the scale was confirmed, a confirmatory factor analysis was conducted. The reliability of the extracted scales was verified with the Cronbach's Alpha reliability coefficient. The analysis of differences between two groups and a continuous variable was performed using the following tests: Mann-Whitney U. Cramer's V test was used to determine the strength of relationships.

The adopted significance level was *p*=.05, indicating the occurrence of statistically significant correlations or differences [56, 57].

### Characteristics of the participants

The studies were conducted in a group of 670 respondents. The study group included 465 (69.4%) nurses and 205 (30.6%) midwives. The mean age of the study subjects was 41, whereby 256 (38.2%) of the subjects were between 41 to 50, and the least subjects, i.e. 115 (17.2%) between 31 and 40 years of age. Almost 60% of the respondents work at the conservative department. The majority of the subjects, i.e. 252 (37.5%) are characterised by a five-year or shorter seniority at their current place of work. A vast majority of the subjects, i.e. 653 (97.5%) work on a full-time basis. The most, i.e. ²/_3_ of the respondents have a university degree, where 234 (34,9%) have a bachelor’s degree in nursing/midwifery, and 196 (29.3%) have a master’s degree in nursing/midwifery. In order to classify the results in the context of completed education, two groups were distinguished:

- I – subjects with higher education, including higher vocational education
- II – subjects with vocational education (licensed nurse, licensed midwife)
- Table 1 presents the study group characteristics with regard to socio-demographic variables and educational-organisational variables.

**Table 1:** Characteristics of the study group

**Characteristic variables**		**n**	**%**
Age (in years)	< 30	159	23.7
	31—40	115	17.2
	41—50	256	38.2
	> 51	140	20.9
Profession	nurse	465	69.4
	midwife	205	30.6
Type of education	vocational	235	35.1
	higher	435	64.9
Department	treating	270	40.3
	conservative	400	59.7
Seniority at current place of work (in years)	< 5	252	37.5
	6 -10	105	17.8
	11 -15	131	15.7
	16—20	77	11.5
	> 21	117	17.5
Amount of working time	full time	653	97.5
	part time	7	1.0
	over full time	10	1.5

## Results

### Reliability and structural relevance of the Belimage scale

In determining the theoretical relevance of the Belimage scale, Exploratory Factor Analysis (EFA) with Varimax rotation was used, which allows for minimizing the number of variables with high factor loadings through orthogonal rotation. This simplifies the interpretation of factors. In order to isolate the internal structure of the scale regarding self-assessment of the professionalism in the specified skills and attitudes consisting of statements, an exploratory analysis was performed using the method of principal axes.

To determine the number of factors, the criterion of eigenvalues was used, which showed that 5 factors should be isolated.

All the factors account for 54.33% of the total score variance.

KMO value was 0.938 and the Bartlett’s sphericity test gave a result of Chi-square = 8354.98; df = 276; *p* < 0.001.

After performing the Varimax rotation with Kaiser normalisation it was shown that the first factor: *instrumental and technical skills* is strongly loaded by the statements: keeping medical documentation (individual patient observation record, fever record, report from duty), ability to perform therapeutic and diagnostic procedures (preparing medications, wound care, applying drains, drip administration, blood sampling, etc.), ability to provide basic care (personal hygiene, feeding, mobilisation, etc.), technical skills related to operation of equipment (monitors, pumps, etc.), and logistic skills (equipment set up, supplies, etc.). Their respective loadings were: 0.69; 0.64; 0.62; 0.55; 0.53. As a result of the analysis it was shown that the value of Cronbach’s alpha statistics for 5 items was 0.843. The achieved result is high and indicates reliability of the measuring instrument.

The second factor: *behaviours and attitudes* is strongly loaded by the statements: taking responsibility for the care provided, professional bedside manner (empathy, undertaking ethical issues, etc.), care (taking care of, looking after), critical attitude (to procedures and decisions made in the hospital, to doctors’ decisions, to the patient's/family's questions, etc.), reformulation skills (explaining the words of the doctor to the patient and their family, and vice versa). Their respective loadings were: 0.67; 0.59; 0.58; 0.48; 0.38. The value of Cronbach's alpha statistics for 5 items was 0.829. The achieved result is high and indicates the reliability of the measuring instrument.

The third factor: *intellectual and cognitive skills* is strongly loaded by the statements: creativity (finding alternative solutions), use of knowledge (using previous experiences in order to solve a current problem), scientific approach (updating knowledge, implementing study results in the professional practice), flexibility (adjusting to a given situational context), consistency in providing nursing care (observation, interpretation, planning, assessment).

Their respective loadings were: 0.71; 0.69; 0.55; 0.53; 0.47. The value of Cronbach's alpha statistics for 5 items was 0.845. The achieved result is high and indicates reliability of the measuring instrument.

The fourth factor: *social and communication skills* is strongly loaded by the statements: communicating with the family, communicating with the patient, communicating with peer nurses/midwives, communication with peer paramedics, educating/informing (communicating information, instructions, knowledge, etc. to the patient and their family). Their respective loadings were: 0.71; 0.70; 0.57; 0.44; 0.41. As a result of the analysis it is noticed that the value of Cronbach’s alpha statistics for 5 items was 0.806. The achieved result is high and indicates reliability of the measuring instrument.

The fifth factor: *organisational skills* is strongly loaded by the statements: administrative skills (telephone, referral notes for tests, ordering medications, administration forms, etc.), organisation of patient care, delegating nursing tasks to other subjects (students, cleaners), organisation of work in cooperation with other healthcare professionals.

Their respective loadings were: 0.68, 0.42; 0.41; 0.37. The value of Cronbach's alpha statistics for the four items was 0.730. The achieved result is high and indicates reliability of the measuring instrument.

### Professional image of nurses and midwives

The surveyed nurses and midwives were asked to determine the extent to which they perceive their professionalism in the above-mentioned skills and approach and the extent to which, in their opinion, a nurse/midwife must have mastered the above-mentioned skills and approach.

The analysis conducted with the use of the Mann–Whitney-U test shows that in the real image, the ability of creative and critical thinking and acting is more important for nurses (M = 4.13; SD = 0.45) than in the group of midwives (M = 4.02; SD = 0.42), and the differences are statistically significant U = 39,067.50; *p* < 0.001. There is also a tendency that shows that innovativeness and practice based on scientific evidence is more significant in the group of nurses (M = 3.99; SD = 0.52) than in the group of midwives (M = 3.80; SD = 0.55) U = 37,450.50; *p* < 0.001 (Table 2).

**Table 2:** Self-image of nurses and midwives from a behavioral perspective

The nursing/midwifery profession is:		n	M	SD	U	*p*
Instrumental skills Documentation and organisation of care	nurse	465	4.25	0.49	42,833.00	.034
	midwife	205	4.20	0.47		
Care and ethical attitudes towards the patient	nurse	465	4.16	0.49	47,172.50	.828
	midwife	205	4.17	0.46		
Ability of creative and critical thinking and acting	nurse	465	4.13	0.45	39,067.50	.000
	midwife	205	4.02	0.42		
Communication skills	nurse	465	4.19	0.48	47,159.50	.821
	midwife	205	4.20	0.42		
Innovativeness and practice based on scientific evidence	nurse	465	3.99	0.52	37,450.50	.000
	midwife	205	3.80	0.55		

*M* Mean, *SD* Standard deviation, *U* Mann–Whitney-U test, *p* Statistical significance level

The respondents were asked to express their opinion on the perception of the nurse and midwife profession by society (projection) and in relation to their own profession (introspection). For this purpose, 10 statements were distinguished, which are included in Table 3. The respondents could choose more than one option.

**Table 3:** Respondents' perceptions of their own image of a nurse/midwife and society’s image of a nurse/midwife

The scope of perception of the nurse/midwife profession		n	%	Chi-square	df	*p*
The profession of a nurse/midwife is a vocation	own	175	26.1	23.018	1	.000
	social	277	41.3			
A nurse/midwife only follows orders	self	10	1.5	398.913	1	.000
	social	428	63.9			
Being a nurse/midwife means taking care of the patient personal hygiene and preparing dressings	self	47	7.0	183.538	1	.000
	social	299	44.6			
The work of a nurse/midwife is hard	self	442	66.0	314.560	1	.000
	social	49	7.3			
The work of a nurse/midwife is poorly paid	self	445	66.4	331.156	1	.000
	social	43	6.4			
The work of a nurse/midwife is a profession which inspires admiration	self	65	9.7	0.833	1	.000
	social	55	8.2			
The work of a nurse/midwife is a profession involving high responsibility	self	375	56.0	274.985	1	.000
	social	38	5.7			
Nurses/midwives are right hands of doctors	self	88	13.1	13.517	1	.000
	social	144	21.5			
In order to become a nurse/midwife, no long-term education is required	self	6	0.9	247.531	1	.000
	social	265	39.6			
A nurse/midwife performs their work fully independently	self	66	9.9	18.473	1	.000
	social	25	3.7			

*df* Degrees of freedom, *p* Statistical significance level

According to the study on nurses and midwives, society considers the job of a nurse/midwife to be a vocation—277 (41.3%), that nurses/midwives are the right hands of doctors – 144 (21.5%), and that their job is to primarily to follow doctors’ orders – 428 (63.9%), as well as to take care of the personal hygiene of patients and apply dressings – 299 (44.6%). Nurses and midwives claim that according to society, no long-term education is required to become a nurse/midwife – 265 (39.6%).

Analysis has shown that the majority of nurses and midwives consider their job to be hard – 442 (66%) and poorly paid – 445 (66.4%), and that they consider their profession to involve high responsibility– 375 (56%). A small group think that they do their job fully independently – 66 (9.9%), and that their profession inspires admiration – 65 (9.7). The statistical analysis has shown a significant difference in the perception of the above-mentioned aspects of the nurse and midwife profession. The self-image of the profession significantly differs from the social image seen by the respondents (Table 3).

The respondents were asked about their opinion regarding satisfaction from being a nurse/midwife, setting aside the present working conditions in the perspective of self-image and social image. The statistical analysis has demonstrated that the respondents are generally satisfied in the perspective of a hard – 338 (76.5%) and poorly paid job – 337 (75.7%), and the responsibility that they exercise – 268 (71.5%). In the perspective of the social image, the nurses and midwives are satisfied with regard to taking care of the patients’ personal hygiene and doing dressings – 228 (76.3%) and fulfilling their vocation – 207 (74.7%). Statistically significant differences appear in the perspective of the professional self-image, since the nurses/midwives consider their work to be hard (*p* = 0.000), involving high responsibility (*p* = 0.000) and poorly paid (p = 0.001). In the perspective of how the profession is seen by society, statistical differences are revealed in the opinion on vocation (*p* = 0.001) and regarding care of the patients’ personal hygiene and preparing dressings (*p* = 0.002) (Table 4).

**Table 4:** Level of satisfaction with the image of a nurse/midwife in self-perception and the image of a nurse/midwife as perceived by the public

Level of satisfaction with being a nurse/midwife?		**Perception by society**		**Self-perception**					
		n	%	n	%	Chi-square	df	*p*	Cramer's V
The profession of a nurse/midwife is a vocation	very dissatisfied	0	0.0	4	2.3	16.143	3	.001	0.189
	dissatisfied	34	12.3	14	2.3				
	satisfied	207	74.7	148	84.6				
	very satisfied	36	13.0	9	5.1				
A nurse/midwife only follows orders	very dissatisfied	6	1.4	0	0	4.272	3	.234	0.099
	dissatisfied	71	16.6	0	0				
	satisfied	299	69.9	10	100				
	very satisfied	52	12.1	0	0				
Being a nurse/midwife means taking care of a patient’s personal hygiene and preparing dressings	very dissatisfied	1	0.3	2	4.3	14.927	3	.002	0.208
	dissatisfied	30	10.0	11	23.4				
	satisfied	228	76.3	30	63.8				
	very satisfied	40	13.4	4	8.5				
The work of a nurse/midwife is hard	very dissatisfied	0	0.0	3	0.7	18.703	3	.000	0.195
	dissatisfied	17	34.7	59	13.3				
	satisfied	32	65.3	338	76.5				
	very satisfied	0	0.0	42	9.5				
The work of a nurse/midwife is poorly paid	very dissatisfied	0	0.0	0	0.0	13.705	2	.001	0.168
	dissatisfied	14	32.6	56	12.6				
	satisfied	23	53.5	337	75.7				
	very satisfied	6	14.0	52	11.7				
The work of a nurse/midwife is a profession which inspires admiration	very dissatisfied	2	3.6	2	3.1	9.868	3	.020	0.287
	dissatisfied	1	1.8	10	15.4				
	satisfied	49	89.1	44	67.7				
	very satisfied	3	5.5	9	13.8				
The work of a nurse/midwife is a profession involving high responsibility	very dissatisfied	2	5.3	2	0.5	19.365	3	.000	0.217
	dissatisfied	3	7.9	58	15.5				
	satisfied	21	55.3	268	71.5				
	very satisfied	12	31.6	47	12.5				
Nurses/midwives are the right hands of doctors	very dissatisfied	0	0.0	4	4.5	10.265	3	.016	0.210
	dissatisfied	11	7.6	10	11.4				
	satisfied	108	75.0	53	60.2				
	very satisfied	25	17.4	21	23.9				
In order to become a nurse/midwife, no long-term education is required	very dissatisfied	0	0.0	0	0.0	0.316	2	.854	0.034
	dissatisfied	39	14.7	1	16.7				
	satisfied	213	80.4	5	83.3				
	very satisfied	13	4.9	0	0.0				
A nurse/midwife performs their work fully independently	very dissatisfied	0	0.0	2	3.0	6.031	3	.110	0.257
	dissatisfied	0	0.0	9	13.6				
	satisfied	20	80.0	49	74.2				
	very satisfied	5	20.0	6	9.1				

*df *Degrees of freedom, *p*: statistical significance level

## Discussion

Nursing is a specialty about which stereotypes have persisted throughout history and is constantly seen as a female profession that is subordinated to the position of a doctor and deprived of its own competence. All of this leads to the image of the nursing profession that differs from reality, constituting a real, significant and acute problem that prevents professional development and has a direct impact on social trust, resource allocation and quality of care, as well as earnings and professional satisfaction [58].

For a long time, nurses have lived in a dualistic structure, in which the path to professionalism is restricted by nursing stereotypes in society. This duality of the nursing world has led to a mismatch between the individual and the environment, which can lead to nurses' job dissatisfaction and low productivity. Therefore, research on the image of nurses/midwives is justified, and the search for and adaptation of research tools that meet the measurement reliability criteria is expected.

Reliability and structural validity of the Belimage scale. Currently, there is no data available in the literature describing the international adaptation of the Belimage Scale [53].

### Self-image of nurses and midwives

In the study group, independence in making important decisions on the care of patients and in their own work is much more often mentioned by nurses. It is difficult to clearly establish if nowadays midwives are able to make independent decisions in the Polish health care system. In theory, certainly so. The results of the conducted studies may be related to the midwives’ place of work, i.e. level 3 facilities, where a vast majority of patients are women with a complicated course of pregnancy. Nurses working in outpatient care have a more positive self-image than those working at stationary facilities [59].

The study shows that nurses pay attention to health problems and potential complications in a patient much more often (*p* = 0.025). Competencies given by the act on the profession of nurse and midwife concentrate on recognising needs and nursing problems, planning and exercising nursing care of a patient, independent provision of preventive, diagnostic, therapeutic and rehabilitation services in a specified scope, as well as performing medical rescue operations, education and health promotion (Act on the profession of nurse and midwife). Own studies show that midwives more often act according to instructions, procedures, rules, principles and protocols. This is to a large extent caused by the medicalisation of midwifery. The proportion of hospital deliveries is very high and has not changed for years. Midwives much less frequently (in practice) are able to diagnose medical problems of patients under their care. The American Nurses Association (ANA) defines the scope of a nurse and midwife practice as protection, promotion and optimisation of health and abilities; prevention of illness and injury; alleviation of suffering; and advocacy in the care of individuals, families and populations. This shows independence of the profession and its exceptional effect on patient welfare, not only in health care institutions, but also in patient populations (ANA 2022).

The study subjects have a generally good opinion on the cooperation in the therapeutic team and their own creativity; they have enough opportunities to discuss with other nurses/midwives the problems related to nursing care they encounter in practice (*p* = 0.002). In this respect, satisfaction of nurses is much higher than that of midwives. Literature confirms that a positive working environment and department character has a material impact on work satisfaction, involvement and quality of care [60, 61]. An effective management of a team requires integrity, competence and atmosphere of mutual trust. Such teams are capable of achieving high quality and effectiveness at their place of work [52].

The results suggest that there is a discrepancy in the image of the nursing profession between nurses and society, which may contribute to a non-adjustment of a nurse to society. The results have also confirmed a negative correlation of non-adjustment with job satisfaction and results of a nurse’s work [1].

### Public image of nurses and midwives

This image is partly created by nurses, due to their invisibility and lack of public discourse. Nurses derive their self-assessment and professional identity from the public image, work environment, work value, education and traditional social and cultural values. In comparison to other studies [62] participants of this study have assessed a higher public image. It is assumed that nurses in this country feel appreciated by society more and more often. Nurses assess the public image with regard to interpersonal strength (factor 1) and interpersonal skills (factor 3), the latter confirming the claim that nurses are appreciated by their trusting character, but often there is limited public understanding of their professionalism and competencies [28].

They found that nurses in Upper Valais have a good self-image and evaluate their public image more negatively than their own. The assumption was confirmed that the self-image of study participants from various institutions differs from their assessment of their own social image. This discrepancy differs slightly from the results of other international studies in which PNIS or a similar tool was also used to record the image of nurses [63].

Nurses and midwives play a key role in the implementation of complex, interdisciplinary treatment plans, and support other members of a health care team, providing an effective coordination of care. It is a false and misleading concept that nurses’/midwives’ basic usefulness is to follow doctors’ orders [61]. Numerous studies conducted on a group of nurses indicate that a decisive factor in implementing the autonomy of the profession expressed by taking important decisions regarding patient care and their own work, is the attitude of co-workers [60, 64]. When midwives and doctors working in the same environment cooperate and share their style of practice and skills, patients may fluently move between one and the other service provider according to need, and at the same time supplement and improve work, and contribute to much fewer medical interventions [24]. In a therapeutic team, it is necessary to develop cooperation based on trust, good communication and knowledge of competencies committed to safety and quality of care [65]. The factors affecting an inconsistent image of nurses included: diversity of education/references, image seen as secondary, lack of leadership development, lack of professionalism, images in the media and Internet, personal experiences of patients, treatment of nurses by other employees, and adopted gender roles [53].

Own studies have demonstrated that nurses and midwives control their scope of professional duties (*p* = *0.006*). Professional values expressed by protectiveness and justice (taking responsibility for fulfilling health need of a culturally diversified population) are currently considered one of the basic duties of nurses with regard to patient care [2]. It seems that proper bedside manner is disturbed by numerous duties, such as keeping more and more extensive medical records with an increasing number of patients per staff member. Studies of other authors confirm that the multitude of tasks and insufficient number of staff members on duty results in focusing a nurse’s attention on technical, organisational and documentation activities, making it difficult for them to fulfil the protective function (State Long-Term Policy for Nursing and Midwifery in Poland (including works initiated in 2018 and 2019)).

The image of care in society has also been described in a dual form. Nurses notice that society shows them gratitude and respect for being a nurse. On the other hand, academisation of nursing is also widely commented by society: “For many people it is unnecessary to pursue a degree of bachelor, then master, of nursing. “It is absolutely unnecessary”…” [66].

### Evidence application to practice

In the professions of high public trust, such as medical professions, a high level of professional identification is an extremely important element. A person’s identification with the performed profession is a component of professional identity. Together with professionalism, it is an important element that integrates representatives of a given profession. In order to improve their public image and acquire a stronger position in health care organisations, nurses must increase their prominence. This may be accomplished by continuous education and a challenging work environment, which encourages nurses to stand up for themselves. Moreover, nurses should take better advantage of strategic positions, such as manager, nursing educator or clinical specialist nurse, and use their professionalism to show society what their work is really about [52].

A negative image of nurses in the public opinion, and comparing them to doctors and other health care professionals may evoke a sense of inferiority and powerlessness in nurses [67].

It is important to cultivate a positive professional self-assessment, since it aids preserving a good physical and mental state, strengthening of the affirmation of one’s own possibilities, building a positive mind set and self-confidence in future clinical practice, and in turn entices students to better participate in nursing studies in order to ensure a more effective patient care [95]. A positive image of nursing/midwifery will result in a greater interest in education in these fields, reduce the process of professional fluctuation and contribute to ensuring the stability of the nursing and midwifery staff.

Systematic monitoring of nurses' and midwives' own and social image is a condition for implementing reasonable steps to modify this image. Therefore, in the absence of local measurement tools, the adaptation and validation of the Belimage scale is a necessary step in the creation of a minimum research workshop.

### Future directions

In order to improve the public image, but also to preventively further strengthen their self-image, nursing and midwifery staff must have the possibility of multi-level institutional support, starting from a pre-graduate education system, a post-graduate education system, institutions supervising nursing care, scientific societies and the professional self-government of nurses and midwives. Therefore, it seems necessary to develop a strategy to strengthen the image of nurses and midwives in order to attract nursing staff that will meet the health needs of society in the future. As part of the draft strategy, nurses should be made aware that their self-image is reflected in how society sees them and that nurses themselves are crucial in strengthening their own image in society. When changing this image, more attention should be paid to social media, i.e. the Internet, YouTube, Facebook, Tik-Tok. Conducting comparative research analyzing the impact of the Covid-19 pandemic or a significant increase in the remuneration of Polish nurses and midwives will allow us to determine the stability of their own and social perception of the professional image of nurses and midwives.

### Strengths and limitations

Relevant literature does not contain many reports on the studies of a professional image conducted among Polish nurses and midwives, making it difficult to comprise a comprehensive and comparative presentation of the issue. Both the current and predicted demographic situation in these professional groups, as well as a regularly growing disproportion between the demand for nursing and midwife care (aging society) and possibilities of providing such care (staff shortage), makes it necessary to indicate, by means of empirical research, measures to increase interest in the profession of a nurse and midwife. As mentioned in the introduction, one such method is to improve the social image of nurses and midwives. Therefore, the presented studies help to indicate factors related to the improvement of the image of professions that are experiencing shortages.

Moreover, studies conducted by Angel have revealed significant differences in the understanding of the professional self-image of nurses, whose probable cause is cultural heterogeneity and a high level of collectivist ideologies [96]. This fact, especially in reference to the ideology remembered from the recent past, may be significant in the shaping of the professional image of the Polish nurse and midwife.

Despite the limitations, it was possible to achieve an initial insight into the self-assessment of qualified nurses and midwives, and their perception of the public image. The results suggest that nurses and midwives have a positive self-image in spite of the limited or distorted public understanding of their professionalism and competencies.

## Conclusions

1. The Polish adaptation of the Belimage scale has achieved acceptable reliability values. The theoretical structure of the tool is five-factor (instrumental and technical skills, behaviors and attitudes, intellectual and cognitive skills, social and communication skills, organizational skills).
2. The study found differences in nurses' and midwives' images of creative and critical thinking and acting, as well as innovation and evidence-based practice.
3. The analysis of the results showed differences in nurses' and midwives' self- and social perceptions of their professions in each of the scopes analyzed. The biggest discrepancies concern the professional self-perception of nurses/midwives.
4. The level of satisfaction with one's own and the society’s perception of the image of the nursing and midwifery professions varies the most in terms of the opinion that the work of a nurse/midwife is a profession with significant responsibility and hard work.

## Data Availability

No new data were created or analyzed in this study. Data sharing is not applicable to this article.
